# M-atrial natriuretic peptide and nitroglycerin in experimental acute hypertensive heart failure: differential actions of two cGMP activating therapeutics

**DOI:** 10.1186/2050-6511-14-S1-P43

**Published:** 2013-08-29

**Authors:** Paul M McKie, Alessandro Cataliotti, Tomoko Ichiki, S Jeson Sangaralingham, Horng H Chen, John C Burnett

**Affiliations:** 1 Cardiorenal Research Laboratory, Rochester, MN, USA; 2 Division of Cardiovascular Diseases, Rochester, MN, USA; 3 Mayo Clinc and Foundation, Rochester, MN, USA

## Background

Systemic hypertension is a common characteristic in acute heart failure (HF). This increasingly common phenotype is frequently associated with renal dysfunction and requires renal enhancing therapies. In a canine model of HF and acute hypertension we defined the cardiorenal actions of M-atrial natriuretic peptide (MANP), a novel particulate guanylyl cyclase A receptor activator (pGC-A) developed at the Mayo Clinic, and nitroglycerin (NTG), a soluble guanylyl cyclase (sGC) activator. Compared to native ANP, MANP has more sustained renal enhancing, blood pressure lowering and aldosterone inhibiting action.

Based on the expression of the pGC-A receptor in the glomerulus, renal tubule and adrenal gland, compared to the more widespread expression of sGC in the renal vasculature, we hypothesized that MANP would have a more robust effect upon the glomerular filtration rate (GFR) and natriuretic actions and with greater aldosterone suppressing effects than sGC activation with NTG. To test these hypotheses, we used a canine model of combined HF and hypertension. Specifically, HF was induced by chronic (10 days) rapid right ventricular pacing (180 bmp), while hypertension was induced by continuous angiotensin II infusion (on day 11). We than characterized the cardiorenal and humoral actions of intravenous MANP (n=7), NTG (n=7), and vehicle (n=7) infusion**.**

## Results

Mean arterial pressure (MAP) was reduced by both MANP (139±4 to 118±3 mmHg, p<0.05) and NTG (137±3 to 116±4 mmHg, p<0.05); similar findings were observed for reductions in pulmonary wedge pressure with MANP (12±2 to 6±2 mmHg, p<0.05) and NTG (12±1 to 6±1 mmHg, p<0.05). MANP enhanced renal function with significant increases in GFR (38±4 to 53±5 ml/min, p<0.05), renal blood flow (132±18 to 236±23 ml/min, p<0.05), and natriuresis (11±4 to 689±37 mEq/min, p<0.05) with inhibition of aldosterone (32±3 to 23±2 ng/dL, p<0.05), whereas NTG had no significant effect on these renal parameters or aldosterone activation.

## Conclusion

Our results demonstrate differential cardiorenal and adrenal actions of pGC activation with MANP, a novel GC-A activator, compared to sGC stimulation with NTG in experimental acute hypertensive HF. These renal enhancing actions and concomitant aldosterone suppressing effects make MANP, now entering clinical trials, an attractive therapeutic for HF with concomitant hypertension, especially where renal protection is a key therapeutic goal.

